# Use and Evaluation of Generative Artificial Intelligence by Medical Students in Japan

**DOI:** 10.31662/jmaj.2024-0375

**Published:** 2025-07-02

**Authors:** Izuki Amano, Kisho Obi-Nagata, Ayane Ninomiya, Yuki Fujiwara, Noriyuki Koibuchi

**Affiliations:** 1 Department of Integrative Physiology, Gunma University Graduate School of Medicine, Maebashi, Japan

**Keywords:** generative AI, ChatGPT, artificial intelligence, large language model, hallucination

## Abstract

**Introduction:**

Generative artificial intelligence (AI) has become more accessible due to technological advancements. While it can support more efficient learning, improper use may lead to legal issues or hinder self-directed learning. Medical education is no exception, as generative AI has the potential to become a powerful tool. However, its practicality remains uncertain. Therefore, we investigated how generative AI is perceived among medical students and utilized within the realm of medical education.

**Methods:**

In January 2024, we conducted a study with 123 second-year medical students who had completed a physiology course and laboratory training at Gunma University, Japan. Students used ChatGPT (Chat Generative Pre-trained Transformer) 3.5 (OpenAI) for four tasks and evaluated its responses. A survey on the use of generative AI was also conducted. Responses from 117 participants were analyzed, excluding six non-participants.

**Results:**

Among the students, 41.9% had used ChatGPT. The average scores for tasks 1-4 were 6.5, 4.6, 7.4, and 6.2 out of 10, respectively. Although 13% had a negative impression, 54 students found it challenging to apply for medical purposes. However, 64.1% expressed a willingness to continue using generative AI, provided its use extended beyond medical contexts.

**Conclusions:**

Nearly 60% of students had never used generative AI before, which is consistent with general usage trends. Although they were impressed by the speed of generative AI responses, many students found that it lacked precision for medical studies and required additional verification. Limitations of generative AI, such as “hallucinations,” were evident in medical education. It remains important to educate students on AI literacy and their understanding of the potential issues that generative AI could bring about.

## Introduction

In recent years, generative artificial intelligence (AI) has become increasingly familiar due to rapid technological advancements. Notably, in November 2022, OpenAI released ChatGPT (Chat Generative Pre-trained Transformer), an AI chatbot based on a large language model (LLM) primarily specialized in dialogue. ChatGPT generates “human-like” responses by understanding user prompts and providing answers that combine ethical judgment, emotional sensitivity, logical analysis, and creative expression, all without additional training. Its high-quality answers and practical usability are widely recognized ^(1)^.

While generative AI can facilitate efficient learning, it also poses risks of legal violations, infringing on the rights of others, or raising security concerns, depending on the input data and how its output is used. Consequently, there are various opinions on its use in academic fields. For example, some academic journals strictly prohibit the use of LLMs in research papers, while others permit their use if authors disclose the prompts, explicitly state the use of LLMs, and assume responsibility for the content ^(2)^. Although the scientific community is still navigating its stance on generative AI, it is clear that we all need to be skilled in understanding its limitations and appropriate use. Therefore, education that incorporates AI use must be developed. At Gunma University, guidelines for the utilization of generative AI in education were established in December 2023 ^(3)^. These guidelines do not restrict the use of AI in learning but emphasize the appropriate use by reminding that the generated content may violate laws or infringe on the rights of others, depending on the input data and its application.

In medical education, generative AI has been tested against various national medical licensing examinations, such as the United States Medical Licensing Examination ^(4)^, the UK’s Membership of the Royal Colleges of Physicians of the United Kingdom (Part 1) ^(5)^, and Japan’s National Medical Licensing Examination ^(6), (7)^. ChatGPT has surpassed the passing scores of all of them, with newer versions achieving even higher marks. A recent systematic review of these trials reported that GPT (Generative Pre-trained Transformer)-3.5 achieved a passing or comparable score in 38.5% (10/26) of cases, while GPT-4.0 reached this benchmark in 89.7% (26/29) of cases ^(8)^. These results are promising; however, the issue of “hallucinations,” where LLMs generate fabricated or incorrect answers, remains a significant challenge. Although future technological improvements may address this issue, both providers and recipients of medical education should continue to exercise utmost caution regarding these hallucinations and remain aware of the limitations at all times ^(9)^.

How can generative AI be utilized by medical students? Based on previous reports, Hale et al. ^(9)^ classified the potential applications of generative AI in medical education into five categories: “nonclinical learning assistant,” “content developer,” “virtual patient,” “clinical decision-making tutor,” and “medical writing.” However, these possibilities may represent only the beginning of the integration of generative AI into medical education. It is anticipated that, in the future, the personalization of such tools will advance to better meet the needs of individual learners. Also, medical knowledge is a dynamically evolving field, shaped by discoveries in basic medical science, the development of new drugs and therapies, and changes in healthcare policies. A variety of expertise is involved, encompassing basic scientists, clinicians, allied health professionals, statisticians, and policymakers. As such, generative AI must continuously learn from various fields and stay current with the most up-to-date information. Furthermore, considering the ultimate impact on patient care, it is essential to critically assess whether the data provided by generative AI are both accurate and reliable. There is also the issue that ethical guidelines and regulations have not yet caught up with the rapid pace of technological advancement. Medical educators must remain vigilant in ensuring that medical students are aware of these factors when using generative AI ^(10)^.

Despite these advancements, it is unclear how much awareness medical students have of generative AI and its potential for medical education. In this study, second-year medical students at Gunma University were tasked with using generative AI to solve assignments for a physiology lab report. The students evaluated the accuracy of the AI’s output, and a subsequent survey aimed to assess the actual usage of generative AI among medical students.

## Materials and Methods

### Participants

・Study subjects: A total of 123 students who had completed a physiology course and participated in the physiology laboratory training on January 9 and 16, 2024.

・Submission period and method: Students were instructed to submit their reports between January 17 and January 27, 2024.

### Presented assignments

The students were instructed to input the following tasks (tasks 1-4) into ChatGPT-3.5 (OpenAI), evaluate the responses provided, and fact-check them by consulting textbooks and academic papers. They then assessed any discrepancies between ChatGPT’s responses and the model answers.

#### Task 1

The second heart sound (S2) physiologically splits, but it can also indicate various heart diseases. Choose one condition that pathologically causes S2 splitting, explain the mechanism behind the splitting, and describe the available treatment options.

#### Task 2

While S2 can split, the first heart sound (S1) can also sometimes be heard as split. Choose one condition that causes S1 splitting and explain the mechanism behind the splitting, and describe the available treatment options.

#### Task 3

Explain the procedure for measuring blood pressure.

#### Task 4

Based on the illustration (Supplemental Figure 1), what would the blood pressure measurement result be? (Correct the result and compare it with your own measurements taken during the lab).

### Survey questions

The students were asked to answer the following questions, with their explanation written in Japanese:

Q1. Have you ever used ChatGPT before? (Yes or No)

Q2. If you answered “Yes” to Q1, have you ever used ChatGPT for report assignments or similar tasks? (Yes or No)

Q3. What score (out of 10) would you give ChatGPT’s answers to each task? Please explain the reasons for your evaluation for each task.

Q4. What are your thoughts after using ChatGPT for this medical report assignment?

Q5. Would you like to use ChatGPT again in the future? (Yes or No, please explain reasons)

### Data analysis

A simple tabulation was performed for each survey item. For the free-response sections of the survey, the responses were first translated into English using DeepL Translator (DeepL GmbH, Cologne, Germany). These translated responses were then processed using custom-written code in MATLAB R2023b (MathWorks, Natick, MA, USA) for sentiment analysis. This analysis quantified qualitative sentiment data from the free responses on a scale from −1 to 1. Scores were categorized as follows:

- Negative: −1 to −0.6

- Slightly Negative: −0.6 to −0.2

- Neutral: −0.2 to 0.2

- Slightly Positive: 0.2 to 0.6

- Positive: 0.6 to 1.0

All statistical analyses were performed using R (version 4.1.0). Differences between two groups were analyzed using the Wilcoxon signed-rank test. A p-value of <0.05 was considered statistically significant.

### Ethical considerations

Students were informed that their participation and responses to the survey would not affect their grades or evaluations in the lab. Additionally, they were given an opportunity to opt out of the study at any time. The study received approval from the Gunma University Ethics Committee for Human Research (reference number HS2024-015).

## Results

A total of 117 students were included in the analysis, after excluding six students who did not participate in the experiment or complete the survey.

### Prior ChatGPT usage experience

Of the 117 students, 49 students (41.9%) had used ChatGPT before (Table 1-1).

**Table 1.** Students’ Perspectives on ChatGPT Use and Performance Based on the Survey and Sentiment Analysis.

(1) Q1. Have you ever used ChatGPT before?					
	**Yes, n (%)**	**No, n (%)**
Total (n = 117)	49 (41.9)	68 (58.1)
(2) Q2. If you answered “Yes” to Q1, have you ever used ChatGPT for report assignments or similar tasks?					
	**Yes, n (%)**	**No, n (%)**
Total (n = 49)	5 (10.2)	44 (89.8)
(3) Q3. What score (out of 10) would you give ChatGPT’s answers to each task?					
**Tasks**	**positive, n (%)**	**slightly positive, n (%)**	**neutral, n (%)**	**slightly negative, n (%)**	**negative, n (%)**
Task 1	1 (0.9)	7 (6.1)	61 (53.5)	30 (26.3)	15 (13.2)
Task 2	2 (1.7)	10 (8.8)	50 (43.9)	38 (33.3)	14 (12.3)
Task 3	2 (1.7)	18 (15.8)	44 (38.6)	40 (35.1)	10 (8.8)
Task 4	3 (2.6)	12 (10.5)	46 (40.4)	33 (28.9)	20 (17.5)
(4) Q4. What are your thoughts after using ChatGPT for this medical report assignment?					
** Total**	**positive, n (%)**	**slightly positive, n (%)**	**neutral, n (%)**	**slightly negative, n (%)**	**negative, n (%)**
Total (n = 115)	3 (2.6)	12 (10.4)	46 (40.0)	34 (29.6)	20 (17.4)
(5) Q5. Would you like to use ChatGPT again in the future? (Yes or No, please explain reasons.)					
	Yes, n (%)	No, n (%)
Total (n = 117)	75 (64.1)	42 (35.9)
(6) Sentiment analysis of the free-response section in Q5					
**Total**	**positive, n (%)**	**slightly positive, n (%)**	**neutral, n (%)**	**slightly negative, n (%)**	**negative, n (%)**
Total (n = 113)	33 (29.2)	35 (31.0)	17 (15.0)	20 (17.7)	8 (7.1)

(1) Results of the survey on prior ChatGPT usage, (2) Experience of using ChatGPT for academic assignments. A survey was conducted with 49 respondents who answered “Yes” to Q1, (3) The results of analyzing the free-text responses for each task (task 1-4) using sentiment analysis. Analysis was conducted for 114 participants, excluding the three who did not provide responses. (4) The results of sentiment analysis on the free-text survey responses regarding whether students would like to use ChatGPT for medical report assignments. Analysis was conducted for 115 participants, excluding the two who did not provide responses. (5) Willingness to use ChatGPT in the future. Survey results in Yes or No format, (6) The results of sentiment analysis on free-text survey responses are presented. Analysis was conducted for 113 participants, excluding the four who did not provide responses. ChatGPT: Chat Generative Pre-trained Transformer; Q: question.

### Prior use of ChatGPT for academic assignments

Of the 49 students who had used ChatGPT before, five (10.2%) reported having used ChatGPT for university assignments, such as reports (Table 1-2).

### Medical students’ evaluation of ChatGPT’s performance on medical assignments

**Figure 1.**
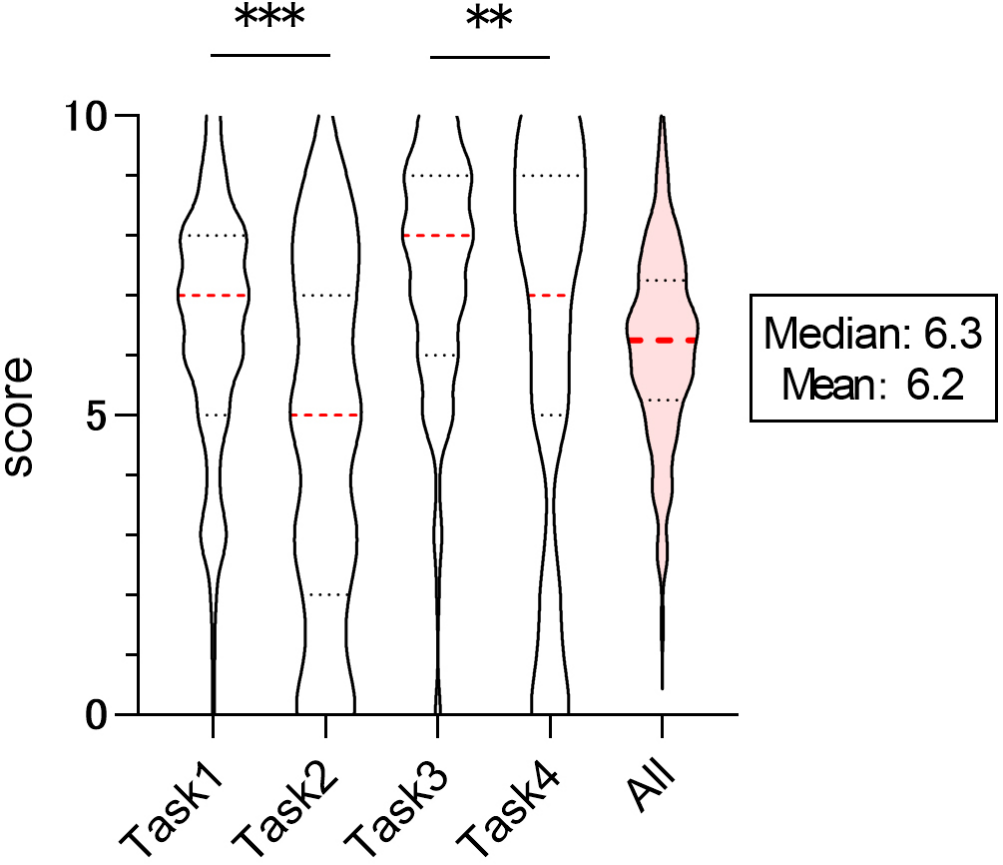
Evaluation of ChatGPT’s responses to medical assignments by medical students. A violin plot illustrates the results of scoring ChatGPT’s performance on each of the four individual tasks and the overall assignments, rated on a 10-point scale. **p < 0.01, ***p < 0.001 determined by Wilcoxon’s signed rank test.

The students evaluated ChatGPT’s responses to each task on a 10-point scale. The median and mean scores were as follows: task 1: 7.0 and 6.5, task 2: 5.0 and 4.6, task 3: 8.0 and 7.4, task 4: 7.0 and 6.2. The overall median and mean score across all tasks were 6.3 and 6.2, respectively. While there was variation in the evaluations depending on the task, the overall evaluation of ChatGPT’s performance in solving the tasks was around six points. When comparing the ratings across tasks within the same category (task 1 vs. task 2, task 3 vs. task 4), the score for task 2 was significantly lower than that for task 1 (p = 0.000000104), and similarly, the score for task 4 was significantly lower than that for task 3 (p = 0.00679) (Figure 1). Additionally, sentiment analysis was conducted based on free-response comments regarding ChatGPT’s answers to each task. For task 1, 7.0% of students had a positive or slightly positive impression, while 39.5% had a negative or slightly negative impression. For task 2, 10.5% had a positive or slightly positive impression, while 45.6% had a negative or slightly negative impression. For task 3, 17.5% had a positive or slightly positive impression, while 43.9% had a negative or slightly negative impression. For task 4, 13.2% had a positive or slightly positive impression, while 46.5% had a negative or slightly negative impression. In summary, around 10% of the students were satisfied with ChatGPT’s responses, while approximately 40% expressed dissatisfaction (Table 1-3).

### Opinions on using ChatGPT for medical reports

When surveyed about the use of ChatGPT for medical report writing, 13.0% of students expressed a positive or slightly positive perspective, whereas 47.0% conveyed a negative or slightly negative viewpoint (Table 1-4). Several respondents noted that verifying the accuracy of ChatGPT’s outputs was a time-consuming process, which, in some cases, completing assignments independently a more efficient approach.

### Willingness to use ChatGPT in the future

The aggregated results of “Yes” or “No” responses regarding future ChatGPT usage (Table 1-5) and the sentiment analysis of the free-response section (Table 1-6) indicate that more than 60% of students expressed a positive outlook on using ChatGPT in the future (Q5: 75 students, 64.1%; Sentiment analysis of Q5: 68 students, 60.2%). Although the feasibility of using ChatGPT for medical reports remains uncertain (as highlighted in Table 1-4), students generally appreciated the tool’s quick responses, accuracy, and potential applications in other fields, such as language learning and beyond.

## Discussion

A recent survey conducted by Gesellschaft für Konsumforschung (GfK) Japan in November and December 2023 among the general populations in Japan, the United Kingdom, the United States, and India revealed that the recognition of ChatGPT in Japan was 62%, compared to 83% in the United States, 89% in the United Kingdom, and 95% in India. Furthermore, only 33% of Japanese respondents had experience using ChatGPT, whereas usage rates were significantly higher in the United States (77%), the United Kingdom (76%), and India (95%). Among different generations, 69% of Gen Z and 63% of Gen X in Japan were aware of ChatGPT, implying a lower permeation of ChatGPT in Japan compared to the other developed countries ^(11)^. In this study, 41.9% of medical students reported prior experience using ChatGPT (Table 1-1), which somewhat aligns with these global trends. Another survey conducted in June 2023 found that Japan ranked third in global traffic to ChatGPT (7.0%), trailing only the United States (10.2%) and India (8.7%), with usage predominantly from individuals in their 20s to 40s ^(12)^. These discrepancies highlight how familiarity and experience with ChatGPT can vary depending on the timing of surveys. Nonetheless, the data from this study did not significantly deviate from broader trends.

Only 13% of students in this study had a positive impression of using ChatGPT for medical assignments (Table 1-4). One reason for this dissatisfaction was that students found it time-consuming to verify ChatGPT’s responses. Many believed that it would be faster to consult authoritative sources themselves. Since ChatGPT is trained on a mixture of existing texts, documents, and unreviewed online content, there is a risk that some of the information may be incorrect or inconsistent with established knowledge, especially in the medical field. Additionally, the model used in this survey, GPT-3.5, was trained on data up until September 2021, meaning its medical knowledge was not up-to-date.

Takahashi et al. ^(13)^ conducted a study using GPT-4 to create clinical case models, which were then evaluated by physicians. They found that major errors were rare and the quality of information was generally acceptable. However, there was a considerable variation depending on the case, suggesting that more accuracy is needed when using AI-generated content in educational settings. While ChatGPT has the potential to assist in medical education, it is not yet a perfect tool for such purposes. Regardless of how much AI models improve, it will always be essential to scrutinize the validity of their output.

Although ChatGPT remains an imperfect tool, the process of verifying its responses can serve as a valuable learning opportunity for students. Currently, AI should be viewed primarily as a supplemental learning tool rather than a comprehensive solution. Students must recognize the duality that AI can facilitate certain aspects of the learning process; however, it cannot yet replace all aspects of learning. Furthermore, a study by Kazley et al. ^(14)^ found that many students considered it acceptable to use AI to prepare for exams or papers but, they also viewed it unethical to use it to complete assignments. This underlying ethical concern may have contributed to the generally negative evaluation of ChatGPT in this study.

For the tasks related to heart sound auscultation, the score for task 2 was significantly lower than that for task 1. Similarly, for the tasks related to blood pressure measurement, the score for task 4 was significantly lower than that for task 3. The complexity of these tasks likely contributed to the reduced scores. In task 2, the correct answer was “not applicable,” while task 4 required students to input prompts after translating image content into text, leading to varied prompts depending on the individual. According to a systematic review on the application of ChatGPT in the medical field, Fatima et al. ^(15)^ reported that ChatGPT could achieve a 60%-70% accuracy range on simpler, frequently asked questions and pass various medical exams. However, when questions became more complex or new scenarios were introduced midway through a problem, the accuracy dropped significantly. This is consistent with the current study’s finding that hallucinations were more common in tasks 2 and 4, leading to lower evaluations (Figure 1 and Table 1-3 and 1-4).

While many students negatively perceived the use of ChatGPT for medical-related tasks (Table 1-4), more than 60% expressed a positive outlook on its future use (Table 1-5 and 1-6). Those with positive responses expected to use ChatGPT for language learning, generating ideas, or creating hypotheses in a conversational format. Although opinions are still divided when it comes to medical education, we need to keep evaluating its capabilities and limitations by practically using generative AI. As AI becomes more integrated into everyday tools like smartphones and computers, educators and students must understand its potential to fully benefit from its capabilities.

There are several limitations to this study. The sample size was limited to a single institution, country, and academic year, suggesting that the results may not be representative of a larger population of medical students. Additionally, cultural differences between countries make it difficult to draw direct international comparisons. Furthermore, this study only used ChatGPT and did not compare it to other LLMs. Moreover, this study did not evaluate the prompts students used to obtain responses from generative AI. Given that the quality of prompts and the frequency of interactions can significantly influence the responses generated by such systems, the evaluation of prompts represents an important avenue for future research. As a result, it may become essential in medical education to provide students with opportunities to learn how to craft effective prompts that facilitate the generation of desired responses.

As of January 2024, the rate of ChatGPT use among medical students was 41.9%, consistent with usage rates in the general population. Although AI tools like ChatGPT may be useful in general contexts, they do not necessarily meet the high standards required in educational and medical settings. While the tasks in this study did not require the latest medical knowledge, students expressed frustration with the uncertainty and lack of reliable sources in ChatGPT’s responses. Hallucinations, fabrications, and inaccurate explanations remain common in AI-generated content, limiting its potential as a learning tool. These issues are inherent to LLMs, and their use in the medical field must be approached with caution. At the same time, the process of verifying AI-generated content provides a valuable learning opportunity for students. It is crucial to recognize both sides of generative AI: it can support learning but cannot yet fully replace traditional educational methods.

## Article Information

### Conflicts of Interest

None

### Author Contributions

Izuki Amano

Conceptualization, data curation, formal analysis, and wrote manuscript.

Kisho Obi-Nagata

Conceptualization, data curation, formal analysis, and wrote manuscript.

Ayane Ninomiya

Data curation, and reviewed manuscript.

Yuki Fujiwara

Data curation, and reviewed manuscript.

Noriyuki Koibuchi

Supervision of project and review and editing manuscript.

Izuki Amano and Kisho Obi-Nagata contributed equally to this work.

### Approval by Institutional Review Board (IRB)

The study received approval from the Gunma University Ethics Committee for Human Research (reference number HS2024-015).

### Data Availability Statement

Additional data are available from the corresponding author upon reasonable request within 12 months of publication.

## Supplement

Supplemental Figure 1
