# Supplementary material for: Use and Evaluation of Generative Artificial Intelligence by Medical Students in Japan
Source: JMA J. 2025 Jul 2;8(3):730–5. doi: 10.31662/jmaj.2024-0375 (PMC12328371; doi:10.31662/jmaj.2024-0375)
Supplement: Supplemental Figure 1 [file 2433-3298-8-3-0730-s001.pdf]

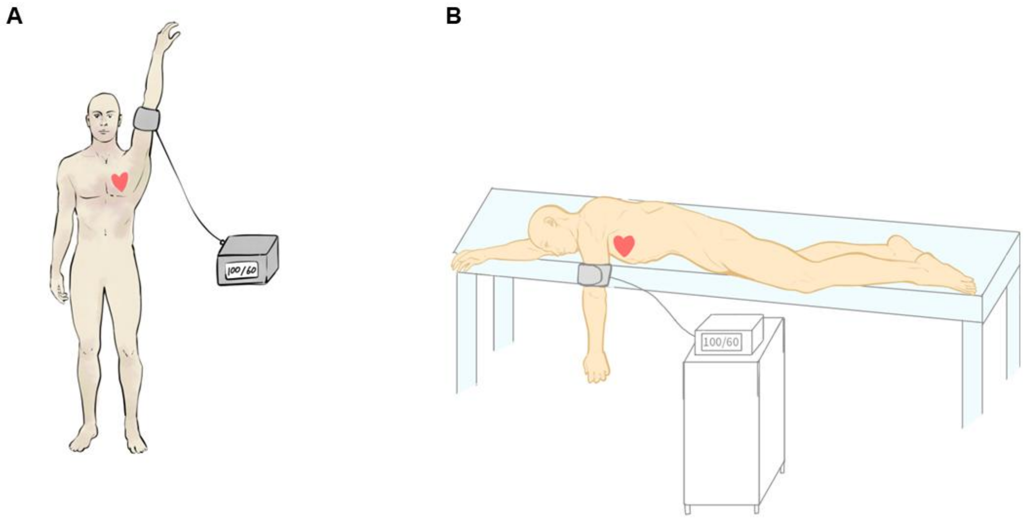

Supplemental Figure 1 Figures presented to students for Task 4. Both A and B depict incorrect methods for measuring blood pressure.
